# Extrapancreatic extension exhibits tumor size-dependent prognostic impact in pancreatic cancer

**DOI:** 10.1038/s41598-026-60797-z

**Published:** 2026-09-15

**Authors:** Jun Zhou, Xiaolin Dou, Wei Wei, Hongqin Jiang, Ling Tang

**Affiliations:** 1 Department of General Surgery, Xiangya Hospital of Central South University, Hunan Province, Changsha, 410008 China; 2 National Clinical Research Center for Geriatric Disorders, Xiangya Hospital of Central South University, Hunan Province, Changsha, 410008 China; 3 Xiangya Nursing School of Central South University, Hunan Province, Changsha, 410008 China; 4 Department of Pharmacy, Xiangya Hospital of Central South University, No. 87 Xiangya Road, Changsha, 410008 Hunan China

**Keywords:** Pancreatic ductal adenocarcinoma, Survival prognosis, Extrapancreatic extension, Tumor size, Interaction effect, Cancer, Oncology

## Abstract

Pancreatic ductal adenocarcinoma (PDAC) remains a highly lethal malignancy requiring precise prognostic stratification. Although extrapancreatic extension (EPE) was removed from the American Joint Committee on Cancer 8th edition staging system in favor of size-based classification, emerging evidence suggests EPE retains prognostic relevance. This population-based study analyzed 22,045 patients with surgically resected PDAC from the Surveillance, Epidemiology, and End Results database (2004–2022) to evaluate EPE’s prognostic significance and its interaction with tumor size. EPE was present in 78.0% of patients and independently predicted worse disease-specific survival (adjusted hazard ratio, 1.29; 95% confidence interval, 1.23–1.35). Notably, the prognostic impact of EPE varied nonlinearly with tumor size, exerting a stronger effect on smaller tumors. A modest but significant negative multiplicative interaction was identified (adjusted hazard ratio, 0.88; 95% confidence interval, 0.79–0.98), suggesting that patients with small EPE-positive tumors exhibited survival comparable to those with larger EPE-negative tumors. These findings were consistent across competing risk analyses. This study demonstrates that EPE remains an important prognostic factor in PDAC, with its effect modulated by tumor size. These results suggest that EPE assessment may warrant consideration in future postoperative staging and risk-stratification frameworks.

**Supplementary Information:**

The online version contains supplementary material available at https://doi.org/10.1038/s41598-026-60797-z.

## Introduction

Pancreatic ductal adenocarcinoma (PDAC) remains one of the most lethal malignancies worldwide, with a 5-year survival rate of approximately 10% despite substantial advances in multimodal treatment^1,2^. This persistently poor prognosis necessitates the critical need for precise staging systems capable of accurately stratifying patients to optimize treatment selection and prognostic assessment^3^. The 7th edition of the American Joint Committee on Cancer (AJCC) TNM staging system emphasizes the biological significance of local invasion, designating extrapancreatic extension (EPE) as the primary determinant for T3 classification^4^. However, this approach has significant limitations. The pancreas lacks a true fibrous capsule, rendering the assessment of extrapancreatic spread inherently subjective and resulting in substantial inter-observer variability among pathologists^5^. Moreover, the detection of peripancreatic soft tissue invasion in over 90% of resected specimens severely compromised EPE’s discriminatory capacity for prognostic stratification^6^. These challenges prompted a paradigm shift in the AJCC 8th edition, which eliminated EPE from the staging criteria entirely and adopted a size-based classification system defining T1 as tumors ≤ 2 cm, T2 as > 2–4 cm, and T3 as > 4 cm^6,7^.

Although this size-centric approach has improved staging reproducibility, accumulating evidence suggests that this modification may inadvertently exclude biologically meaningful prognostic information. While one study reported that EPE lacks independent prognostic value in pancreatic tail tumors^8^, a growing body of literature challenges the adequacy of purely size-based staging by demonstrating EPE’s persistent prognostic relevance^9–12^. Recent investigations have revealed that EPE retains independent prognostic significance even among small tumors^13,14^, with EPE-positive tumors ≤ 2 cm exhibiting survival outcomes comparable to larger tumors without extension. Furthermore, comparative analyses of the AJCC 8th edition versus the Japan Pancreas Society (JPS) staging system, which retained EPE as a staging criterion, consistently demonstrated superior prognostic stratification with EPE-based classification, particularly among patients receiving chemoradiotherapy^11^. These conflicting findings indicate a complex, potentially nonlinear relationship between EPE and tumor size that is inadequately captured by current staging systems. Comprehensive research examining the interaction between EPE and tumor size remains limited.

Given these critical knowledge gaps, we conducted a comprehensive population-based analysis to evaluate the prognostic significance of EPE in PDAC patients. This study aimed to address three key objectives: first, to quantify the independent prognostic impact of EPE on disease-specific survival using both relative and absolute measures; second, to characterize how EPE’s prognostic effect varies across the continuous spectrum of tumor sizes; and third, to formally test for interaction effects between EPE and tumor size that may inform future staging refinements.

## Methods

### Data source and study population

Data for patients diagnosed with pancreatic ductal adenocarcinoma (PDAC; ICD-O-3 codes: 8140, 8500) were extracted from the Surveillance, Epidemiology, and End Results (SEER) database (Incidence—SEER Research Data, 17 Registries, Nov 2024 Sub (2000–2022)) using the National Cancer Institute’s SEER*Stat software (version 9.0.40.1). The inclusion criteria were patients with histological confirmation of PDAC between 2004 and 2022, owing to the availability of EPE and staging information. The exclusion criteria were age < 18 years, unknown demographic, clinicopathological, therapy, or follow-up information, and a follow-up time of < 1 month. To focus on surgically resectable diseases and isolate the independent effects of EPE, we excluded patients with T4 tumors (defined by major vascular involvement), distant metastases (M1), and those who did not undergo surgical resection. These selection criteria yielded a final cohort of 22,045 patients (Supplementary Fig. 1). Given the publicly available and de-identified nature of SEER data, this study was exempted from institutional review board approval at Central South University.

### Variables and definitions

The variables collected in this study included demographic characteristics (age at diagnosis, sex, race, marital status, household income, and county type), clinicopathologic features (year of diagnosis, primary tumor site, grade, tumor size, EPE, and N stage), therapy regimen (surgery, radiotherapy, and chemotherapy), and follow-up data (survival months and cause of death). Notably, EPE was defined as tumor extension beyond the pancreatic parenchyma into surrounding tissues or organs and was harmonized across three SEER coding eras: the Collaborative Stage (CS) Extension field (2004–2015), the SEER-derived AJCC 7th edition T classification (2016–2017; T1–T2 as EPE-negative and T3 as EPE-positive, as 7th edition T3 is defined by extrapancreatic extension), and the EOD Primary Tumor field (2018–2022). Nodal staging adhered to the American Joint Committee on Cancer (AJCC) 8th edition TNM classification system. The primary endpoint was disease-specific survival (DSS), defined as the time from the initial diagnosis to death from pancreatic cancer or the last follow-up, whichever occurred first.

### Statistical analysis

Survival curves were used to characterize the survival probability among patients with and without EPE. Unadjusted survival curves were generated using the Kaplan–Meier method, whereas adjusted survival curves employed direct covariate adjustment based on a multivariable Cox model^15,16^. The effect of EPE on DSS was quantified using relative (hazard ratios) and absolute measures (survival differences). Hazard ratios (HRs) with 95% confidence intervals (CIs) were estimated using Cox models, while absolute survival differences were calculated through survival probability differences at clinically relevant time points (1, 3, 5, and 10 years), and visualized using risk-difference curves to facilitate clinical interpretation^17,18^.

To evaluate whether the effect of EPE varied across tumor sizes, HRs for EPE across tumor sizes were obtained from a three-knot restricted cubic spline (RCS) Cox model^19^. The knots for the RCS models were strategically placed at the 10th, 50th, and 90th percentiles of tumor size distribution. To visualize the absolute effect of EPE on the 3-year DSS across tumor sizes, both dependence and partial dependence plots were generated based on predictions from the multivariate Cox model^20,21^. Dependence plots display the unadjusted overall trend of the predicted survival rate, while partial dependence plots illustrate the adjusted association after accounting for all other covariates in the multivariable model, reflecting the corrected effect on survival. The nonlinearity of the association was assessed using the Wald test.

To investigate the interaction effect between EPE and tumor size, tumor size was dichotomized at 20 mm based on the potential cutoff in RCS curves, in which the hazard ratio for EPE peaked among small tumors and stabilized at approximately 20 mm, and it coincides with the AJCC 8th edition T1/T2 boundary (2 cm). Interaction effects were evaluated on both the multiplicative and additive scales^22,23^. Multiplicative interaction assesses whether the combined effect differs from the product of individual effects, which is calculated as HR₁₁/(HR₁₀ × HR₀₁), where HR₁₁ represents patients with both EPE and tumor size above 20 mm. HR₁₀ represents EPE with tumors ≤ 20 mm and HR₀₁ represents no EPE with tumors > 20 mm. Additive interaction, measured by the relative excess risk due to interaction (RERI = HR₁₁—HR₁₀—HR₀₁ + 1), quantifies whether the combined effect exceeds the sum of individual effects. The 95% CIs for the RERI were calculated using the delta method^24^.

To assess the robustness of the findings, we conducted comprehensive sensitivity analyses using Fine-Gray models, treating death from causes other than pancreatic cancer as a competing event. This approach estimates the cumulative incidence function rather than the cause-specific hazard, thereby providing complementary insights into the prognostic impact of EPE in the presence of competing risks. We performed additional sensitivity analyses for the interaction: (1) stratified by treatment era (2004–2010 versus 2011–2022, dichotomized at the introduction of modern multi-agent chemotherapy); (2) restricted to patients without neoadjuvant therapy, identified using the SEER systemic-therapy-and-surgery sequence; and (3) using an alternative 40-mm threshold (the AJCC 8th edition T2/T3 boundary).

Summary statistics were provided as total frequencies and percentages for categorical variables, while median values with interquartile ranges or means and standard deviations (SD) were reported for continuous variables, as appropriate. Differences in data distribution between groups for categorical and continuous variables were assessed using the χ2 and Mann–Whitney U tests, respectively. Multicollinearity among model covariates was assessed using variance inflation factors, and no meaningful collinearity was detected (all values < 2). All statistical analyses were performed using R software (version 4.4.3). Two-sided P-values < 0.05 were considered statistically significant. Significance levels are denoted as follows: ***p < 0.001, **p < 0.01, and *p < 0.05.

## Results

### Patient characteristics

The study cohort comprised 22,045 patients with surgically resected PDAC (Supplementary Fig. 1), of whom 17,195 (78.0%) exhibited EPE and 4,850 (22.0%) did not (Table 1). The demographic characteristics showed a comparable median age between groups at 68 years (p = 0.923). However, the EPE group demonstrated a slightly higher proportion of male patients (51.1% vs. 47.6%; p < 0.001) and white patients (82.7% vs. 81.0%; p = 0.007). Clinicopathologically, patients with EPE harbored significantly larger tumors (median 32 mm vs. 26 mm; p < 0.001), with a progressive increase in EPE prevalence across tumor size categories (Supplementary Fig. 2): 16.5% in tumors ≤ 5 mm, 35.8% in 5–10 mm, 64.6% in 10–20 mm, 79.7% in 20–40 mm, and 86.5% in tumors > 40 mm. Tumors with EPE more frequently originated from the pancreatic head (76.2% vs. 65.0%; p < 0.001), exhibited poorly differentiated or undifferentiated histology (grade III/IV: 31.3% vs. 22.8%; p < 0.001), and demonstrated more advanced nodal involvement (N2 stage: 28.4% vs. 11.7%; p < 0.001). Treatment patterns differed significantly, with EPE patients undergoing more Whipple procedures (72.3% vs. 64.8%; p < 0.001) and receiving adjuvant radiotherapy and chemotherapy more frequently (29.4% vs. 21.8% and 76.5% vs. 72.7%, respectively; both p < 0.001). Despite more aggressive multimodal therapy, patients with EPE experienced a shorter median follow-up duration (19.0 vs. 23.0 months; p < 0.001) and higher pancreatic cancer-specific mortality (70.1% vs. 51.8%; p < 0.001).

**Table 1:** Patient characteristics stratified by extrapancreatic extension in pancreatic ductal adenocarcinoma.

Variable	Overall N = 22,045^1^	No EPE N = 4,850^1^	EPE N = 17,195^1^	p-value^2^
Age (year)	68.00 (60.00, 75.00)	68.00 (60.00, 75.00)	68.00 (60.00, 75.00)	0.923
Sex				
Female	10,954 (49.7%)	2,541 (52.4%)	8,413 (48.9%)	< 0.001
Male	11,091 (50.3%)	2,309 (47.6%)	8,782 (51.1%)	
Race				
White	18,156 (82.4%)	3,928 (81.0%)	14,228 (82.7%)	0.007
Black	2,018 (9.2%)	496 (10.2%)	1,522 (8.9%)	
Other/Unknown	1,871 (8.5%)	426 (8.8%)	1,445 (8.4%)	
Marital status				
Married	13,924 (63.2%)	3,063 (63.2%)	10,861 (63.2%)	0.971
Single	2,725 (12.4%)	596 (12.3%)	2,129 (12.4%)	
Divorced/Widowed/Separated	4,852 (22.0%)	1,075 (22.2%)	3,777 (22.0%)	
Unknown	544 (2.5%)	116 (2.4%)	428 (2.5%)	
Household income				
Low	5,334 (24.2%)	1,143 (23.6%)	4,191 (24.4%)	0.452
Middle	8,544 (38.8%)	1,882 (38.8%)	6,662 (38.7%)	
High	8,167 (37.0%)	1,825 (37.6%)	6,342 (36.9%)	
County type				
Urban	19,641 (89.1%)	4,353 (89.8%)	15,288 (88.9%)	0.096
Rural	2,404 (10.9%)	497 (10.2%)	1,907 (11.1%)	
Year of diagnosis				
2004–2010	6,106 (27.7%)	1,334 (27.5%)	4,772 (27.8%)	< 0.001
2011–2017	8,497 (38.5%)	1,333 (27.5%)	7,164 (41.7%)	
2018–2022	7,442 (33.8%)	2,183 (45.0%)	5,259 (30.6%)	
Primary tumor site				
Head	16,246 (73.7%)	3,152 (65.0%)	13,094 (76.2%)	< 0.001
Body	1,716 (7.8%)	594 (12.2%)	1,122 (6.5%)	
Tail	2,285 (10.4%)	630 (13.0%)	1,655 (9.6%)	
Others(overlapping/unknown)	1,798 (8.2%)	474 (9.8%)	1,324 (7.7%)	
Grade				
Grade I/II	12,214 (55.4%)	2,773 (57.2%)	9,441 (54.9%)	< 0.001
Grade III/IV	6,491 (29.4%)	1,107 (22.8%)	5,384 (31.3%)	
Unknown	3,340 (15.2%)	970 (20.0%)	2,370 (13.8%)	
Tumor size (mm)	30.00 (24.00, 40.00)	26.00 (20.00, 35.00)	32.00 (25.00, 40.00)	< 0.001
Tumor size category (mm)				
(0,20]	3,573 (16.2%)	1,435 (29.6%)	2,138 (12.4%)	< 0.001
(20,40]	13,599 (61.7%)	2,757 (56.8%)	10,842 (63.1%)	
(40,90 +]	4,873 (22.1%)	658 (13.6%)	4,215 (24.5%)	
N stage				
N0	7,997 (36.3%)	2,848 (58.7%)	5,149 (29.9%)	< 0.001
N1	8,595 (39.0%)	1,434 (29.6%)	7,161 (41.6%)	
N2	5,453 (24.7%)	568 (11.7%)	4,885 (28.4%)	
Surgery				
Partial pancreatectomy	3,030 (13.7%)	978 (20.2%)	2,052 (11.9%)	< 0.001
Whipple procedure	15,578 (70.7%)	3,144 (64.8%)	12,434 (72.3%)	
Total or extended pancreatectomy	3,437 (15.6%)	728 (15.0%)	2,709 (15.8%)	
Radiotherapy				
No	15,843 (71.9%)	3,773 (77.8%)	12,070 (70.2%)	< 0.001
Beam	6,121 (27.8%)	1,058 (21.8%)	5,063 (29.4%)	
Other Radiation	81 (0.4%)	19 (0.4%)	62 (0.4%)	
Chemotherapy				
No	5,363 (24.3%)	1,326 (27.3%)	4,037 (23.5%)	< 0.001
Yes	16,682 (75.7%)	3,524 (72.7%)	13,158 (76.5%)	
Follow-up (months)	20.00 (10.00, 37.00)	23.00 (12.00, 44.00)	19.00 (10.00, 35.00)	< 0.001
Cause of deaths				
Alive	5,758 (26.1%)	1,904 (39.3%)	3,854 (22.4%)	< 0.001
Pancreas cancer	14,575 (66.1%)	2,514 (51.8%)	12,061 (70.1%)	
Other causes	1,712 (7.8%)	432 (8.9%)	1,280 (7.4%)	

^1^Median (Q1, Q3); n (%)

^2^Wilcoxon rank sum test; Pearson’s Chi-squared test.

### Effect of EPE on DSS

**Fig. 1:**
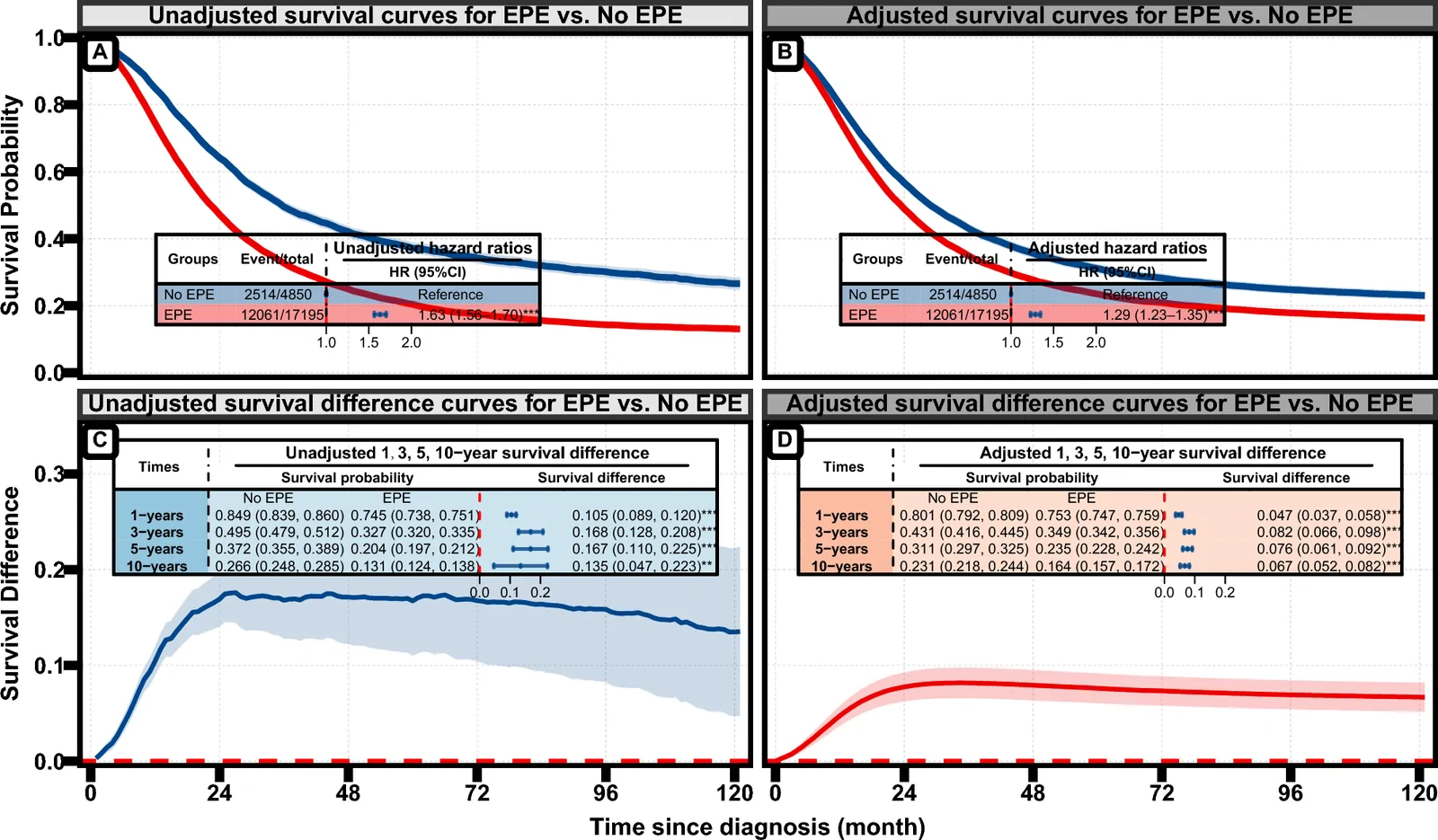
Survival curves and difference curves between EPE groups based on the Cox model. Survival curves (**A**), unadjusted; (**B**), adjusted and survival difference curves (**C**), unadjusted; (**D**), adjusted comparing patients with and without EPE. The adjusted curve was derived from a multivariate Cox model adjusted for age, sex, race/ethnicity, marital status, household income, county type, year of diagnosis, primary tumor size, grade, N stage, surgery, radiotherapy, and chemotherapy. ***p < 0.001, **p < 0.01, and *p < 0.05.

In the Kaplan–Meier analysis (Fig. 1A), disease-specific survival (DSS) declined sharply for EPE patients, while the decline for No EPE patients was more modest (unadjusted HR: 1.63, 95% CI: 1.56–1.70). After adjustment for covariates in multivariate analysis (Fig. 1B), although the decline in DSS for EPE patients was less steep than that observed in the Kaplan–Meier curve, EPE patients still had worse DSS compared to No EPE patients, with an adjusted HR of 1.29 (95% CI: 1.23–1.35). Furthermore, we examined the absolute survival differences between the EPE groups. Patients with EPE consistently presented significantly unadjusted survival differences compared to those without EPE over time (Fig. 1C). This finding was confirmed by the adjusted curves based on the multivariate Cox model (Fig. 1D). Specifically, the unadjusted 1-year, 3-year, 5-year, and 10-year survival differences between EPE and No EPE patients were 10.5% (95% CI: 8.9%-12.0%), 16.8% (95% CI: 12.8%-20.8%), 16.7% (95% CI: 11.0%-22.5%), and 13.5% (95% CI: 4.7%-22.3%), respectively. After adjustment for confounders, absolute survival differences persisted at 4.7% (95% CI: 3.7%-5.8%), 8.2% (95% CI: 6.6%-9.8%), 7.6% (95% CI: 6.1%-9.2%), and 6.7% (95% CI: 5.2%-8.2%) at 1, 3, 5, and 10 years, respectively. Notably, competing risk analysis using Fine-Gray models corroborated these findings (Supplementary Fig. 3). The unadjusted and adjusted hazard ratios for EPE were 1.58 (95% CI: 1.52–1.65) and 1.34 (95% CI: 1.28–1.40), respectively.

### Effect of EPE across tumor size

**Fig. 2:**
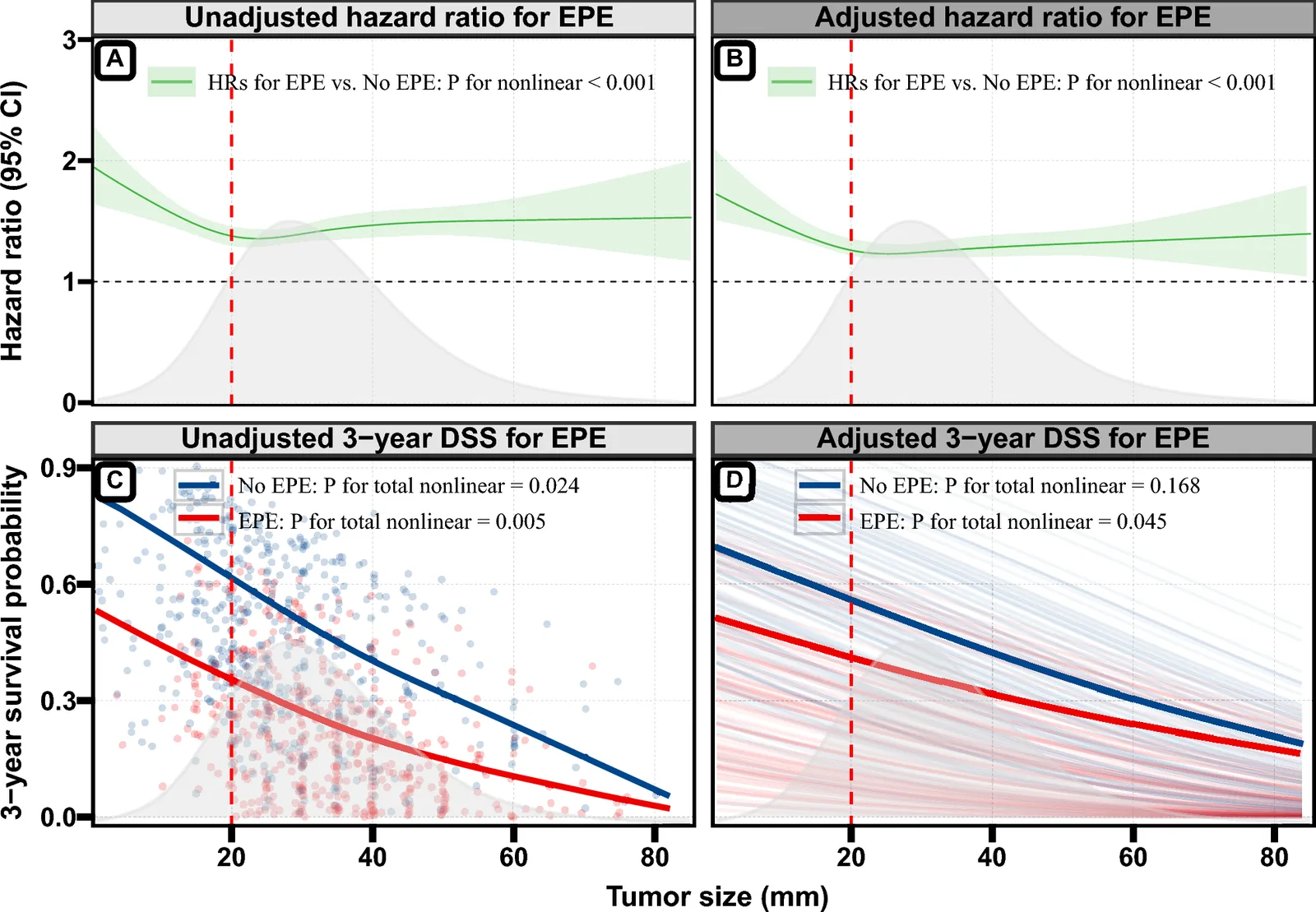
Relative and absolute effects of EPE across tumor sizes based on the Cox model. Restricted cubic spline curves (**A**), unadjusted; (**B**), adjusted illustrating hazard ratios for EPE versus No EPE across tumor size. Dependency and partial dependency plots (**C**), unadjusted; (**D**), adjusted showing 3-year survival probability for EPE and No EPE across tumor size. The gray shaded areas represent the density distribution of tumor size.

Restricted cubic spline analysis revealed a significant nonlinear relationship between tumor size and EPE’s prognostic impact (p for nonlinearity < 0.001; Fig. 2A,B). The hazard ratios for EPE versus No EPE demonstrated a decreasing trend with increasing tumor size, with higher relative risks in smaller tumors that gradually declined and stabilized after approximately 20 mm in both unadjusted (Fig. 2A) and adjusted analyses (Fig. 2B). Dependency and partial dependency plots confirmed that while survival declined with increasing tumor size in both groups, patients with EPE consistently exhibited lower survival across the entire spectrum, with the most pronounced difference in tumors < 20 mm (Fig. 2C,D).

**Fig. 3:**
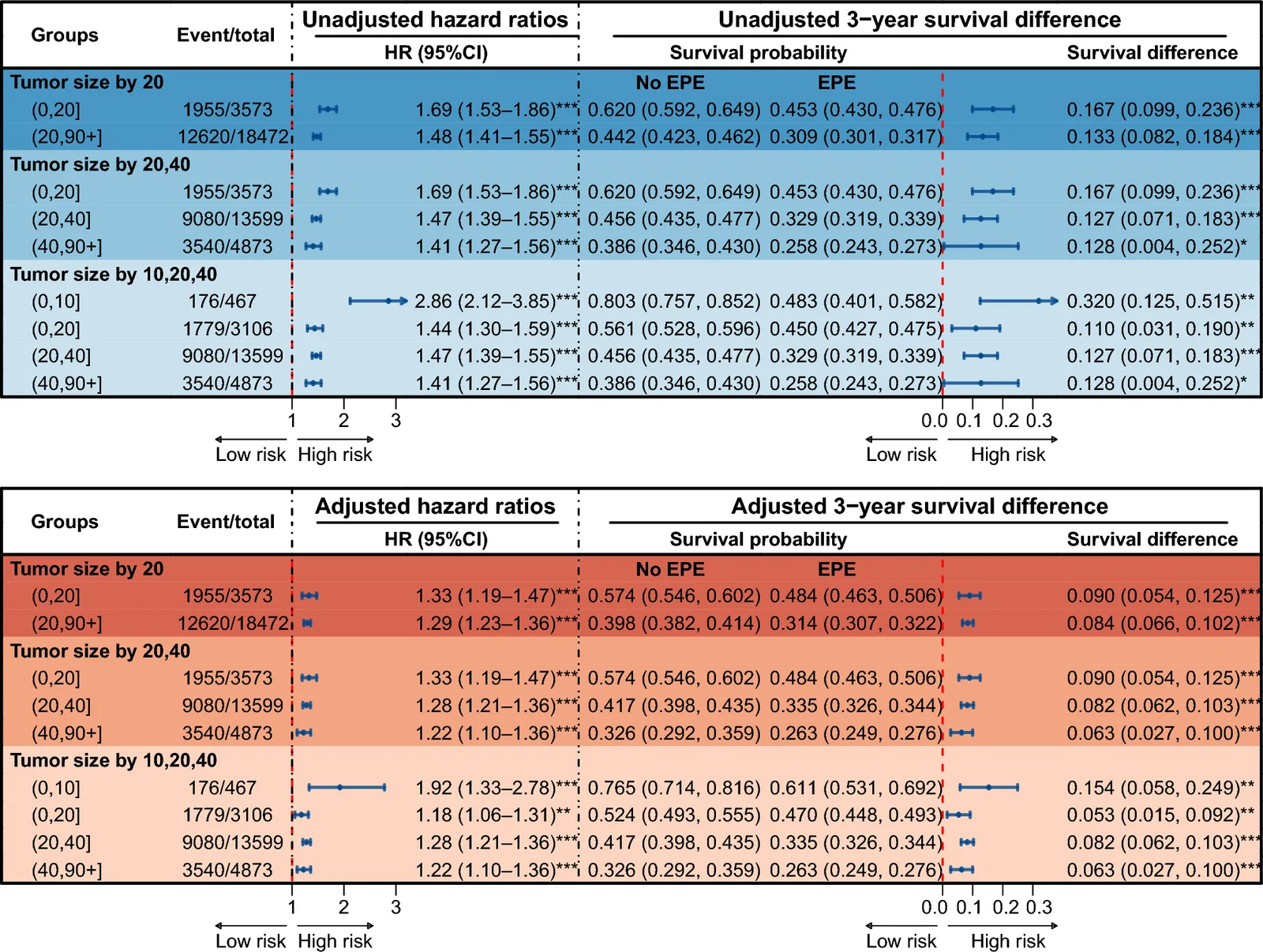
Relative and absolute effects of EPE across different tumor size groups based on the Cox model. Forest plots displaying adjusted hazard ratios (left) and 3-year survival differences (right) for EPE versus no EPE within different tumor size strata. The adjusted analysis was derived from a multivariate Cox model adjusted for age, sex, race/ethnicity, marital status, household income, county type, year of diagnosis, grade, N stage, surgery, radiotherapy, and chemotherapy. ***p < 0.001, **p < 0.01, and *p < 0.05.

Stratified analysis by tumor size further validated these findings (Fig. 3), with EPE’s effect decreasing as the tumor size increased. For tumors 0–20 mm versus 20–90 mm, the adjusted hazard ratios were 1.33 (95% CI: 1.19–1.47) versus 1.29 (95% CI: 1.23–1.36), with corresponding adjusted 3-year survival differences of 9.0% (95% CI: 5.4%-12.5%) versus 8.4% (95% CI: 6.6%-10.2%). Notably, small tumors (0–10 mm) with EPE demonstrated the most extreme impact, with adjusted hazard ratios of 1.92 (95% CI: 1.33–2.78). Competing risk analysis using Fine-Gray models corroborated these findings, confirming EPE’s marked size-dependency with an obvious impact on smaller tumors and stabilization beyond 20 mm (Supplementary Fig. 4).

### Interaction effect between EPE and tumor size

**Fig. 4:**
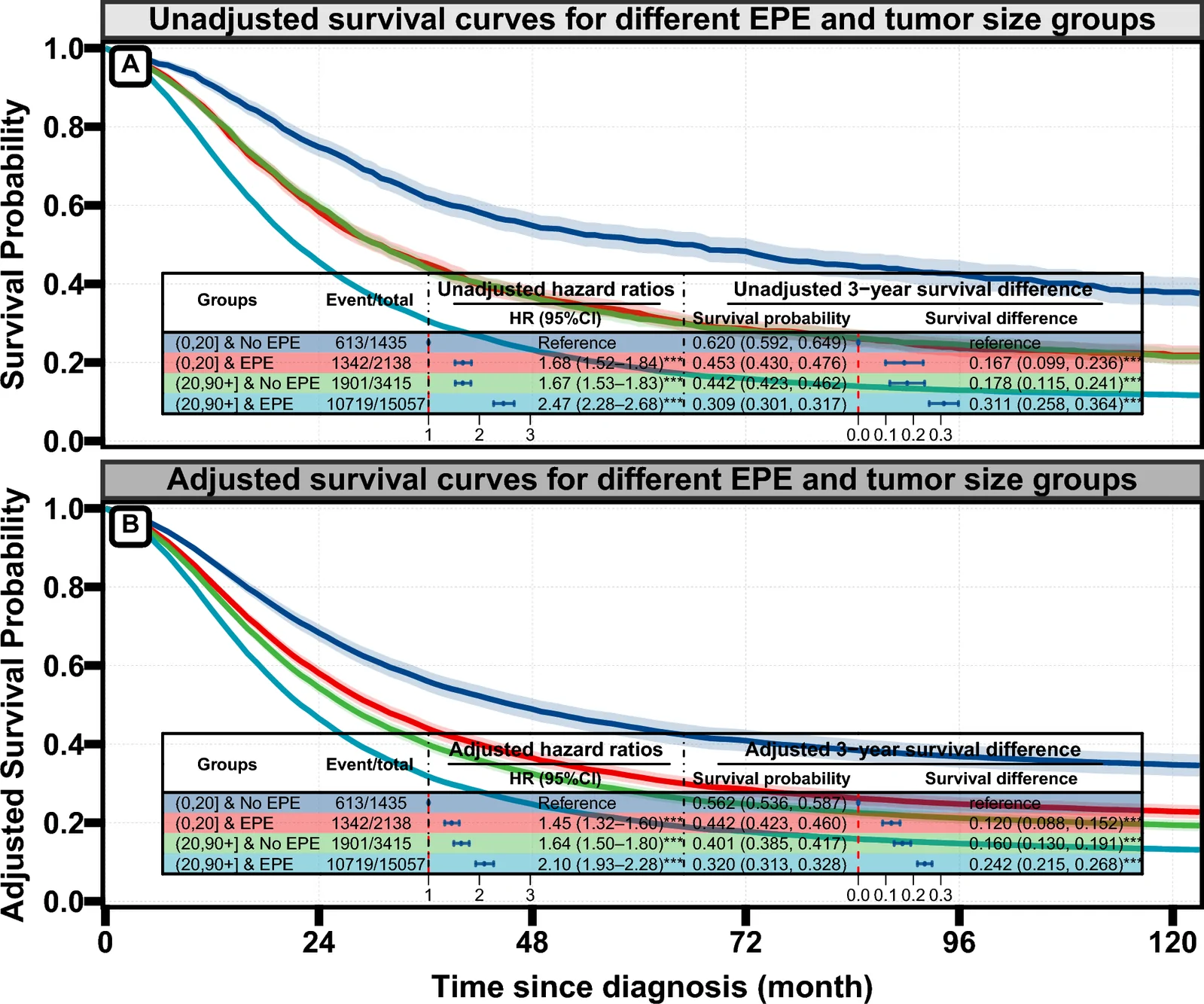
Survival curves for different EPE and tumor size groups based on the Cox model. (**A**) Unadjusted and (**B**) adjusted survival curves for four patient groups stratified by EPE status and tumor size (≤ 20 mm vs. > 20 mm). The adjusted curve was derived from a multivariate Cox model adjusted for age, sex, race/ethnicity, marital status, household income, county type, year of diagnosis, grade, N stage, surgery, radiotherapy, and chemotherapy. ***p < 0.001, **p < 0.01, and *p < 0.05.

To further examine the interaction between EPE and tumor size, patients were divided into four groups: No EPE with tumors ≤ 20 mm, EPE with tumors ≤ 20 mm, No EPE with tumors > 20 mm, and EPE with tumors > 20 mm. Survival analysis (Fig. 4) revealed distinct prognostic patterns among these groups. Using patients with tumors ≤ 20 mm without EPE as the reference, the adjusted hazard ratios were 1.45 (95% CI: 1.32–1.60) for EPE with tumors ≤ 20 mm, 1.64 (95% CI: 1.50–1.80) for no EPE with tumors > 20 mm, and 2.10 (95% CI: 1.93–2.28) for EPE with tumors > 20 mm (Fig. 4, Table 2). Notably, the survival curves for patients with EPE and tumors ≤ 20 mm were closely aligned with those of patients with no EPE and tumors > 20 mm (adjusted hazard ratios 1.45 [95% CI 1.32–1.60] and 1.64 [95% CI 1.50–1.80], respectively), indicating comparable observed survival. This similarity reflects overlapping survival rather than formal statistical equivalence, and the absence of a detected difference should not be interpreted as proof of prognostic equivalence.

**Table 2:** Interaction analysis between different EPE and tumor size groups based on Cox model.

	Unadjusted cox model			Adjusted cox model		
	No EPE	EPE	Effect of EPE within the strata of tumor size	No EPE	EPE	Effect of EPE within the strata of tumor size
	HR [95% CI]	HR [95% CI]	HR [95% CI]	HR [95% CI]	HR [95% CI]	HR [95% CI]
Tumor size: (0,20]	1.00 (Reference)	1.68 (1.52, 1.84)***	1.68 (1.52, 1.84)***	1.00 (Reference)	1.45 (1.32, 1.60)***	1.45 (1.32, 1.60)***
Tumor size: (20,90 +]	1.67 (1.53, 1.83)***	2.47 (2.28, 2.68)***	1.48 (1.41, 1.55)***	1.64 (1.50, 1.80)***	2.10 (1.93, 2.28)***	1.28 (1.21, 1.34)***
Effect of tumor size within the strata of EPE	1.67 (1.53, 1.83)***	1.48 (1.40, 1.56)***		1.64 (1.50, 1.80)***	1.44 (1.36, 1.53)***	
Multiplicative scale	0.88 (0.79, 0.98)*			0.88 (0.79, 0.98)*		
RERI	0.13 (-0.02, 0.27)			0.00 (-0.14, 0.14)		

Multivariable Cox model adjusted for age, sex, race, marital status, household income, County type, year of diagnosis, primary tumor size, grade, N stage, surgery, radiotherapy and chemotherapy

RERI, relative excess risk due to interaction

*** p < 0.001, ** p < 0.01, * p < 0.05

Formal interaction testing on the multiplicative scale demonstrated a significant negative interaction, with a consistent multiplicative term of 0.88 (95% CI: 0.79–0.98; p < 0.05) in both unadjusted and adjusted Cox models (Table 2). This finding indicates that the combined effect of EPE and larger tumor size is less than multiplicative, with EPE’s relative prognostic impact attenuating as tumor size increases. Conversely, the additive interaction was not statistically significant, with an adjusted relative excess risk due to interaction (RERI) of 0.00 (95% CI: -0.14 to 0.14). Competing risk analysis confirmed these results (Supplementary Table 1 and Supplementary Fig. 5). The negative multiplicative interaction was further confirmed in sensitivity analyses stratified by treatment era (Supplementary Table 2), restricted to patients without neoadjuvant therapy (adjusted multiplicative scale 0.84, 95% CI 0.75–0.95; Supplementary Table 3), and using an alternative 40-mm threshold (adjusted 0.85, 95% CI 0.76–0.96; Supplementary Table 4).

## Discussion

This population-based analysis of 22,045 patients with pancreatic ductal adenocarcinoma (PDAC) demonstrated that extrapancreatic extension (EPE) remains a critical independent prognostic factor for disease-specific survival, with its impact significantly modified by tumor size. The adjusted hazard ratio of 1.29 (95% CI: 1.23–1.35) provides quantitative evidence supporting EPE’s prognostic value despite its exclusion from the AJCC 8th edition staging criteria. Critically, the demonstration of a negative multiplicative interaction between EPE and tumor size (multiplicative scale 0.88, 95% CI: 0.79–0.98) reveals that small tumors harboring EPE exhibited survival outcomes comparable to larger tumors without extension, a finding with profound implications for staging refinement. These findings challenge the adequacy of a purely size-based classification and support ongoing debates regarding optimal T-stage stratification in PDAC.

The observed 78% prevalence of EPE, progressively increasing from 16.5% in tumors ≤ 5 mm to 86.5% in tumors > 40 mm, aligns with recent large-scale analyses demonstrating near-universal EPE in resected specimens^13^. Lee et al. reported comparable prevalence rates of 63.2% in T1, 83.4% in T2, and 85.8% in T3 tumors among 37,634 patients in the National Cancer Database, indicating significantly superior median overall survival in EPE-negative versus EPE-positive patients (33.7 versus 20.7 months)^13^. This prevalence is lower than the 91% reported by Saka et al. in resected specimens using standardized grossing protocols, a finding that historically provided the rationale for the AJCC 8th edition’s shift from EPE-based to size-based T-stage classification^6^. However, emerging evidence challenges this paradigm shift. Comparative analyses between the AJCC 8th edition and the Japan Pancreas Society staging system, which retained the EPE criteria, consistently demonstrated superior prognostic discrimination with the EPE-based classification, particularly among patients receiving chemoradiotherapy^11^. Similarly, Kang et al. evaluated the clinical utility of the AJCC 8th edition staging system and highlighted its limitations in accurately stratifying patient prognosis^25^.

The negative multiplicative interaction between EPE and tumor size represents a novel and significant contribution to understanding PDAC biology and staging. This interaction pattern, whereby EPE’s prognostic impact diminishes with increasing tumor size, suggests that small tumors with EPE exhibit more aggressive phenotypes. Our finding that patients with tumors ≤ 20 mm and EPE demonstrated survival curves closely aligned with those having tumors > 20 mm without EPE corroborates previous observations^14^ and extends them through a rigorous statistical analysis. This size-dependent effect of EPE likely reflects fundamental differences in tumor biology, where the early acquisition of invasive capabilities independent of tumor burden signifies an inherently aggressive disease. A recent analysis of 142 patients with small PDAC (≤ 2 cm) found EPE in 78.2% of cases, with EPE presence associated with significantly shorter recurrence-free survival, further supporting the biological significance of EPE in early-stage disease^26^.

At the molecular level, the early acquisition of invasive capacity in small EPE-positive tumors is consistent with reports that drivers of invasion can emerge independently of tumor burden, for example through early KRAS mutant-allele dosage gains that markedly increase metastatic potential^27^ and SMAD4 loss that reshapes tumor-stromal cross-talk to favor local invasion^28,29^. A detailed mechanistic account is beyond the scope of this clinical analysis, and these associations should be regarded as hypotheses requiring dedicated molecular investigation.

From a treatment perspective, our findings suggest that patients with small tumors and EPE may benefit from intensified therapeutic approaches typically reserved for larger tumors. Comparable survival outcomes between small EPE-positive and larger EPE-negative tumors support the consideration of neoadjuvant therapy for technically resectable but biologically aggressive diseases. This aligns with recent clinical trial evidence^30,31^, which demonstrated significant survival benefits for neoadjuvant approaches in borderline resectable disease, with EPE status serving as a key criterion for treatment stratification. The International Study Group of Pancreatic Surgery suggested that biological factors, including EPE status, should guide treatment selection beyond the anatomical resectability criteria^32^. Furthermore, the size-dependent effect of EPE may inform surgical planning, with EPE-positive small tumors potentially warranting more extensive resections and comprehensive lymphadenectomies despite their limited size.

It should be emphasized that EPE is currently a postoperative, pathological diagnosis. Preoperative imaging, including contrast-enhanced CT, MRI, and endoscopic ultrasound, has limited sensitivity for microscopic peripancreatic soft-tissue invasion, and assessment is further constrained by the absence of a true pancreatic capsule and by interobserver variability among pathologists. Accordingly, our findings position EPE primarily as a postoperative prognostic descriptor for refined risk stratification and adjuvant decision-making rather than as a preoperative staging variable under current diagnostic capabilities. Advances in high-resolution imaging, radiomics, and circulating biomarkers may improve the preoperative identification of biologically aggressive small tumors in the future.

Several methodological strengths support this investigation, including an extensive population-based cohort with long-term follow-up, comprehensive multivariable adjustment, and advanced survival modeling techniques incorporating competing risk analyses and restricted cubic splines. The consistency of our findings across both the Cox proportional hazards and Fine-Gray competing risk models with relative (hazard ratios) and absolute measures (survival differences) strengthens the confidence in the observed associations. Nevertheless, several limitations of this study warrant consideration. The retrospective nature of the SEER database analysis introduces the potential for unmeasured confounding despite comprehensive multivariable adjustment. The absence of standardized EPE assessment protocols across contributing institutions may introduce variability in EPE reporting, as highlighted by the Amsterdam International Consensus Meeting^33^. Additionally, the SEER database lacks detailed information on specific EPE patterns, molecular profiling data, and treatment response metrics that could further refine prognostic stratification. Several established prognostic factors in PDAC, including perineural invasion, lymphovascular invasion, preoperative CA 19–9, and ECOG performance status, are not reliably captured in SEER and could not be included. Furthermore, temporal changes in surgical techniques and diagnostic capabilities during the study period (2004–2022) may have influenced the observed outcomes; although SEER records the sequence of systemic therapy relative to surgery, it does not capture regimen, dose, or treatment response, so residual confounding from the non-linear evolution of systemic therapy cannot be fully excluded despite era stratification and adjustment for year of diagnosis. In addition, because EPE remains a postoperative pathological determination, its immediate applicability to preoperative staging is constrained by current imaging limitations.

In conclusion, this large-scale analysis confirmed that EPE remains a critical prognostic factor in pancreatic ductal adenocarcinoma, with its impact modified by tumor size in a complex and nonlinear manner. The observed negative multiplicative interaction indicates that patients with small tumors and EPE have a prognosis comparable to that of patients with larger tumors without EPE. Future staging frameworks may benefit from reincorporating EPE as a postoperative, pathology-based prognostic factor to enhance risk stratification, although prospective validation with standardized assessment is required before clinical application.

## Supplementary Information

Below is the link to the electronic supplementary material.

Supplementary Information

## Data Availability

The datasets used and/or analyzed for the present study are available from the corresponding author on reasonable request.
