# Supplementary material for: Extrapancreatic extension exhibits tumor size-dependent prognostic impact in pancreatic cancer
Source: Sci Rep. 2026 Sep 15;16:28716. doi: 10.1038/s41598-026-60797-z (PMC13578350; doi:10.1038/s41598-026-60797-z)
Supplement: Supplementary file 1 [file 41598_2026_60797_MOESM1_ESM.pdf]

Extrapancreatic Extension Exhibits Tumor Size-Dependent Prognostic Impact in Pancreatic Cancer

Authors:

Jun Zhou, MD [1,2], Xiaolin Dou, MD [1,2], Wei Wei, MD [1,2], Hongqin Jiang, MD[3], Ling Tang, MD [4*]

Affiliations:

1 Department of General Surgery, Xiangya Hospital of Central South University, Changsha 410008, Hunan Province, China

2 National Clinical Research Center for Geriatric Disorders, Xiangya Hospital of Central South University, Changsha 410008, Hunan Province, China

3 Xiangya Nursing School of Central South University, Changsha 410008, Hunan Province, China

4 Department of Pharmacy, Xiangya Hospital of Central South University, Changsha 410008, Hunan Province, China

Supplementary Table 1. Interaction analysis between different EPE and tumor size groups based on Fine-Gray model

	Unadjusted Fine-Gray model			Adjusted Fine-Gray model		
	No EPE	EPE	Effect of EPE within the strata of tumor size	No EPE	EPE	Effect of EPE within the strata of tumor size
	HR [95% CI]	HR [95% CI]	HR [95% CI]	HR [95% CI]	HR [95% CI]	HR [95% CI]
Tumor size: (0,20]	1.00 (Reference)	1.63 (1.49, 1.79)***	1.63 (1.49, 1.79)***	1.00 (Reference)	1.41 (1.28, 1.55)***	1.41 (1.28, 1.55)***
Tumor size: (20,90+]	1.62 (1.48, 1.77)***	2.34 (2.16, 2.53)***	1.44 (1.38, 1.51)***	1.56 (1.43, 1.70)***	1.94 (1.79, 2.11)***	1.24 (1.18, 1.31)***
Effect of tumor size within the strata of EPE	1.62 (1.48, 1.77)***	1.43 (1.35, 1.51)***		1.56 (1.43, 1.70)***	1.38 (1.30, 1.46)***	
Multiplicative scale	0.88 (0.80, 0.98)*			0.88 (0.80, 0.98)*		
RERI	0.09 (-0.05, 0.22)			-0.03 (-0.16, 0.11)		

Note: Multivariable Fine-Gray model adjusted for age, sex, race, marital status, household income, county type, year of diagnosis, primary tumor size, grade, N stage, surgery, radiotherapy and chemotherapy.

Abbreviations: RERI, relative excess risk due to interaction.

Significance levels: *** $p < 0.001$, ** $p < 0.01$, * $p < 0.05$.

Supplementary Table 2. Interaction analysis between different EPE and tumor size groups based on Cox model in patients by year of diagnosis.

	Unadjusted interaction analysis					
	Year of Diagnosis: 2004-2010			Year of Diagnosis: 2011-2022		
	No EPE	EPE	Effect of EPE within the strata of tumor size	No EPE	EPE	Effect of EPE within the strata of tumor size
	HR (95% CI)	HR (95% CI)	HR (95% CI)	HR (95% CI)	HR (95% CI)	HR (95% CI)
Tumor size: (0,20]	1.00 (Reference)	1.61 (1.43, 1.81)***	1.61 (1.43, 1.81)***	1.00 (Reference)	1.79 (1.58, 2.03)***	1.79 (1.58, 2.03)***
Tumor size: (20,90+]	1.67 (1.49, 1.87)***	2.35 (2.12, 2.60)***	1.41 (1.32, 1.50)***	1.83 (1.62, 2.06)***	2.76 (2.48, 3.08)***	1.51 (1.42, 1.61)***
Effect of tumor size within the strata of EPE	1.67 (1.49, 1.87)***	1.46 (1.36, 1.56)***		1.83 (1.62, 2.06)***	1.54 (1.43, 1.66)***	
Multiplicative scale	0.87 (0.79, 0.96)**			0.84 (0.73, 0.97)*		
RERI	0.07 (-0.11, 0.25)			0.14 (-0.05, 0.34)		
	**Adjusted interaction analysis**					
	**Year of Diagnosis: 2004-2010**			**Year of Diagnosis: 2011-2022**		
	No EPE	EPE	Effect of EPE within the strata of tumor size	No EPE	EPE	Effect of EPE within the strata of tumor size
	HR (95% CI)	HR (95% CI)	HR (95% CI)	HR (95% CI)	HR (95% CI)	HR (95% CI)
Tumor size: (0,20]	1.00 (Reference)	1.43 (1.27, 1.61)***	1.43 (1.27, 1.61)***	1.00 (Reference)	1.57 (1.38, 1.78)***	1.57 (1.38, 1.78)***
Tumor size: (20,90+]	1.60 (1.43, 1.80)***	2.03 (1.83, 2.25)***	1.27 (1.18, 1.35)***	1.80 (1.59, 2.03)***	2.31 (2.07, 2.58)***	1.28 (1.20, 1.37)***
Effect of tumor size within the strata of EPE	1.60 (1.43, 1.80)***	1.42 (1.32, 1.52)***		1.80 (1.59, 2.03)***	1.48 (1.37, 1.59)***	
Multiplicative scale	0.89 (0.81, 0.98)*			0.82 (0.71, 0.94)**		
RERI	0.00 (-0.18, 0.17)			-0.06 (-0.25, 0.14)		

Note: multivariable Cox model adjusted for age, sex, race, marital status, household income, county type, primary tumor size, grade, N stage, surgery, radiotherapy and chemotherapy.

Abbreviations: RERI, relative excess risk due to interaction.

Significance levels: *** p < 0.001, ** p < 0.01, * p < 0.05.

Supplementary Table 3. Interaction analysis between different EPE and tumor size groups based on Cox model in patients without neoadjuvant therapy.

	**Unadjusted Cox model**			**Adjusted Cox model**		
	No EPE	EPE	Effect of EPE within the strata of tumor size	No EPE	EPE	Effect of EPE within the strata of tumor size
	HR [95% CI]	HR [95% CI]	HR [95% CI]	HR [95% CI]	HR [95% CI]	HR [95% CI]
Tumor size: (0,20]	1.00 (Reference)	1.72 (1.56, 1.91)***	1.72 (1.56, 1.91)***	1.00 (Reference)	1.52 (1.37, 1.69)***	1.52 (1.37, 1.69)***
Tumor size: (20,90+]	1.85 (1.68, 2.05)***	2.68 (2.45, 2.93)***	1.44 (1.37, 1.52)***	1.74 (1.58, 1.93)***	2.24 (2.04, 2.45)***	1.28 (1.21, 1.36)***
Effect of tumor size within the strata of EPE	1.85 (1.68, 2.05)***	1.55 (1.46, 1.65)***		1.74 (1.58, 1.93)***	1.47 (1.38, 1.57)***	
Multiplicative scale	0.84 (0.75, 0.94)**			0.84 (0.75, 0.95)**		
RERI	0.10 (-0.06, 0.27)			-0.03 (-0.19, 0.13)		

Note: Multivariable Cox model adjusted for age, sex, race, marital status, household income, county type, year of diagnosis, primary tumor size, grade, N stage, surgery, radiotherapy and chemotherapy.

Abbreviations: RERI, relative excess risk due to interaction.

Significance levels: *** p < 0.001, ** p < 0.01, * p < 0.05.

Supplementary Table 4. Interaction analysis between different EPE and tumor size (using 40 mm as cutoff) groups based on Cox model.

	Unadjusted Cox model			Adjusted Cox model		
	No EPE	EPE	Effect of EPE within the strata of tumor size	No EPE	EPE	Effect of EPE within the strata of tumor size
	HR [95% CI]	HR [95% CI]	HR [95% CI]	HR [95% CI]	HR [95% CI]	HR [95% CI]
Tumor size: (0,40]	1.00 (Reference)	1.62 (1.55, 1.70)***	1.62 (1.55, 1.70)***	1.00 (Reference)	1.39 (1.32, 1.46)***	1.39 (1.32, 1.46)***
Tumor size: (40,90+]	1.50 (1.35, 1.66)***	2.12 (2.01, 2.24)***	1.42 (1.28, 1.57)***	1.49 (1.34, 1.66)***	1.76 (1.66, 1.87)***	1.18 (1.07, 1.31)**
Effect of tumor size within the strata of EPE	1.50 (1.35, 1.66)***	1.31 (1.26, 1.36)***		1.49 (1.34, 1.66)***	1.27 (1.22, 1.32)***	
Multiplicative scale	0.87 (0.78, 0.98)*			0.85 (0.76, 0.96)**		
RERI	0.00 (-0.17, 0.18)			-0.11 (-0.28, 0.05)		

Note: Multivariable Cox model adjusted for age, sex, race, marital status, household income, county type, year of diagnosis, primary tumor size, grade, N stage, surgery, radiotherapy and chemotherapy.

Abbreviations: RERI, relative excess risk due to interaction.

Significance levels: *** $p < 0.001$, ** $p < 0.01$, * $p < 0.05$.

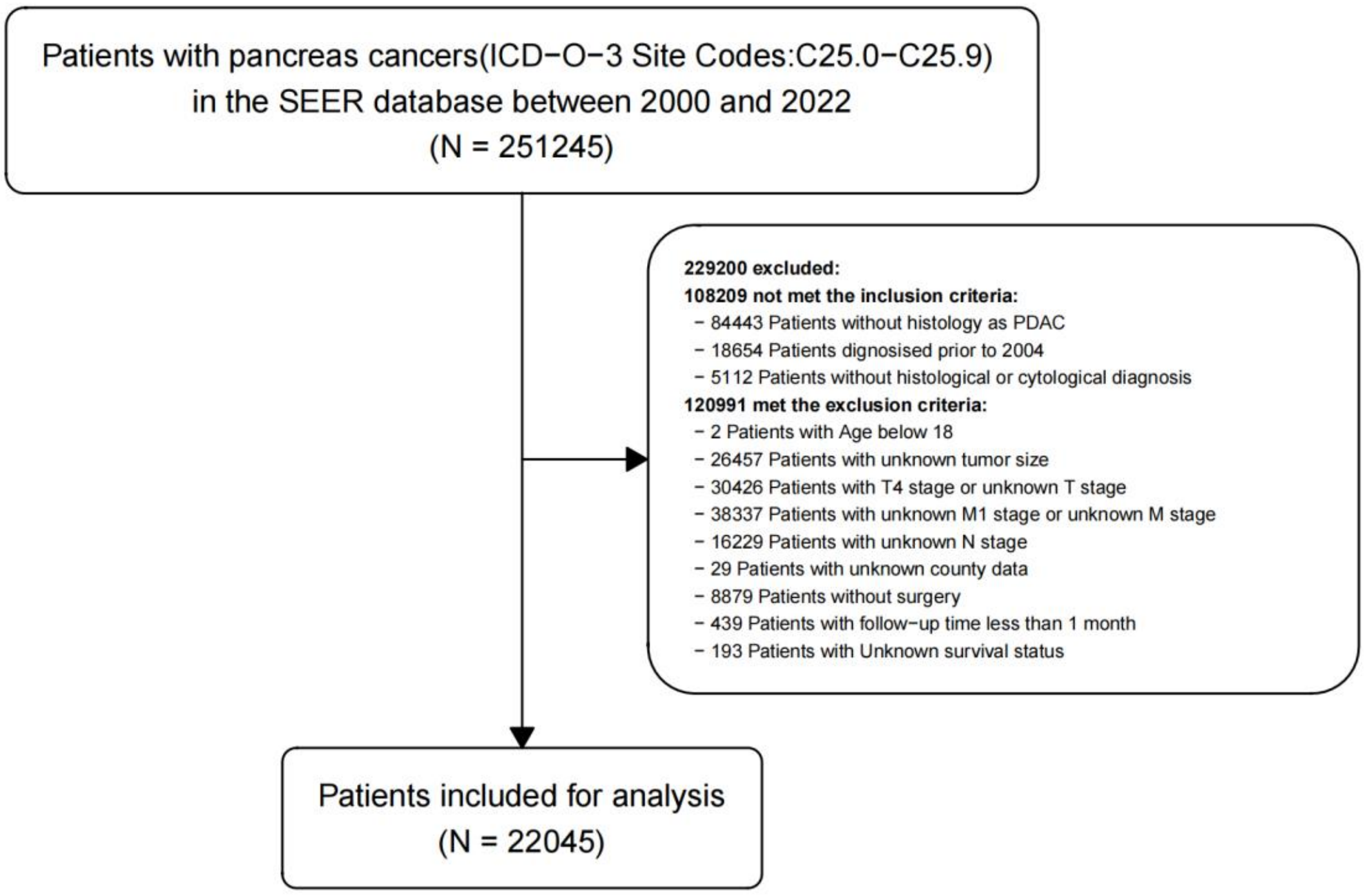

Supplementary Figure 1. Flow diagram of the study patients.

Schematic representation of patient selection from the Surveillance, Epidemiology, and End Results (SEER) database. Patients with pancreatic cancer (ICD-O-3 Site Codes: C25.0–C25.9) diagnosed between 2000 and 2022 were initially identified (N = 251,245). A total of 229,200 patients were excluded based on inclusion criteria (histological confirmation of pancreatic ductal adenocarcinoma, diagnosis between 2004 and 2022) and exclusion criteria (age <18 years, unknown demographic or clinicopathological variables, T4 or M1 disease, no surgical resection, and follow-up <1 month), yielding a final cohort of 22,045 patients.

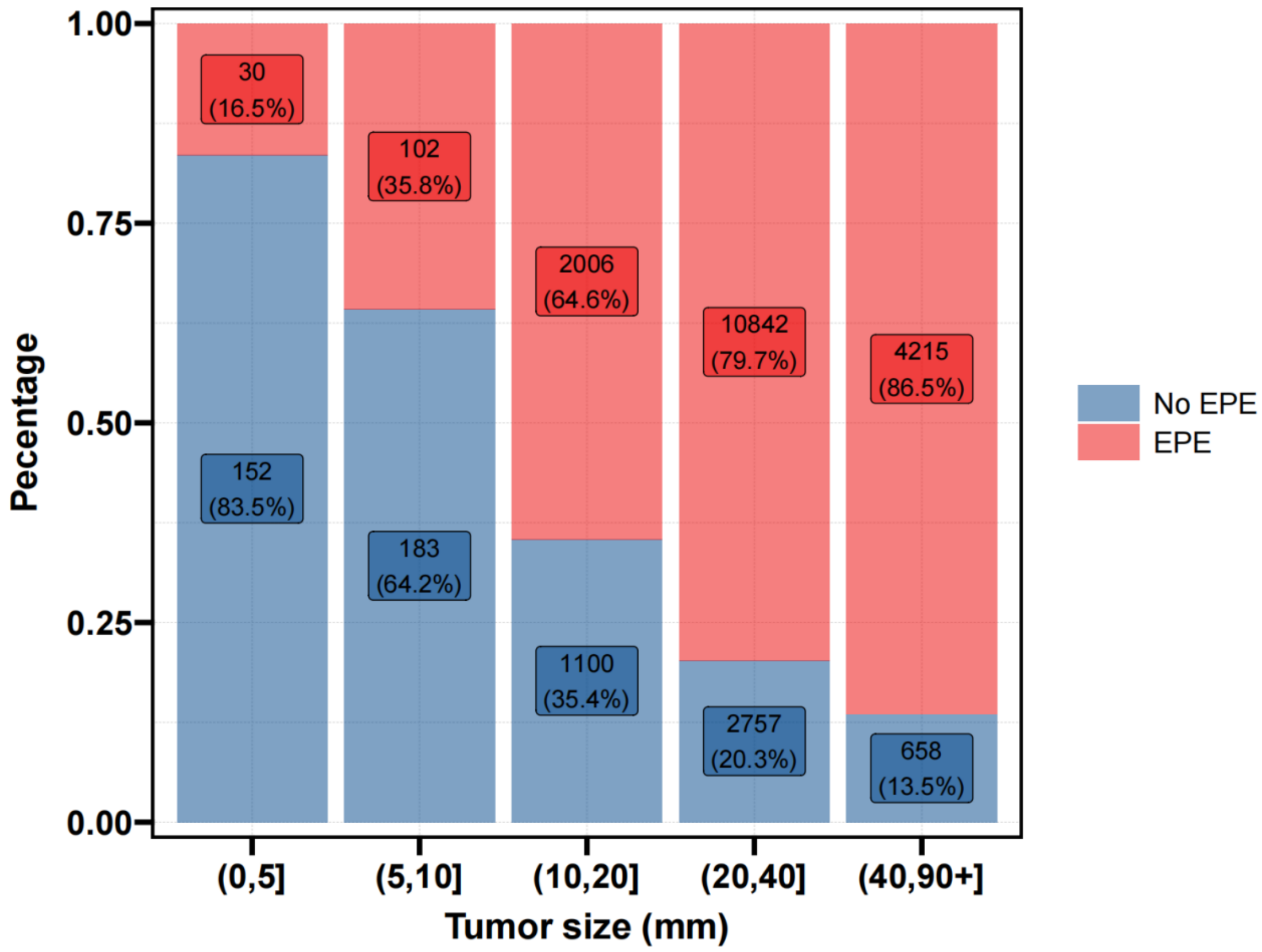

Supplementary Figure 2. Distribution of extrapancreatic extension across different tumor size categories

Stacked bar chart illustrating the prevalence of extrapancreatic extension (EPE) across five tumor size categories: 0–5 mm, 5–10 mm, 10–20 mm, 20–40 mm, and >40 mm. Numbers within bars indicate patient counts; percentages represent the proportion of EPE-positive (red) and EPE-negative (blue) cases within each category.

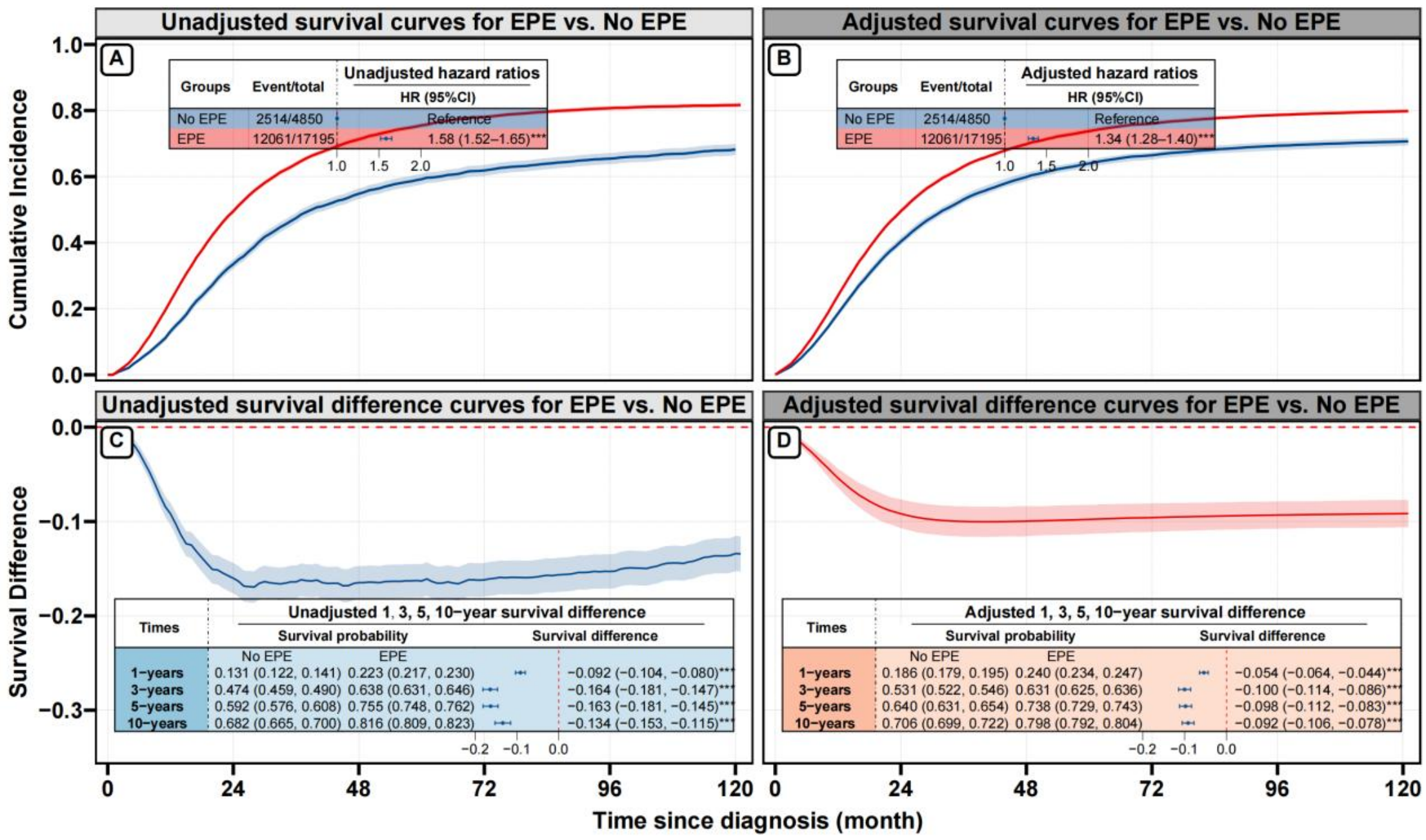

Supplementary Figure 3. Survival curves and difference curves between EPE groups based on the Fine-Gray model.

Cumulative incidence curves (A, unadjusted; B, adjusted) and survival difference curves (C, unadjusted; D, adjusted) comparing patients with and without EPE. The adjusted curve was derived from a multivariate Fine-Gray competing risk model adjusted for age, sex, race/ethnicity, marital status, household income, county type, year of diagnosis, primary tumor size, grade, N stage, surgery, radiotherapy, and chemotherapy. Death from causes other than pancreatic cancer was treated as a competing event. ***p < 0.001, **p < 0.01, and *p < 0.05.

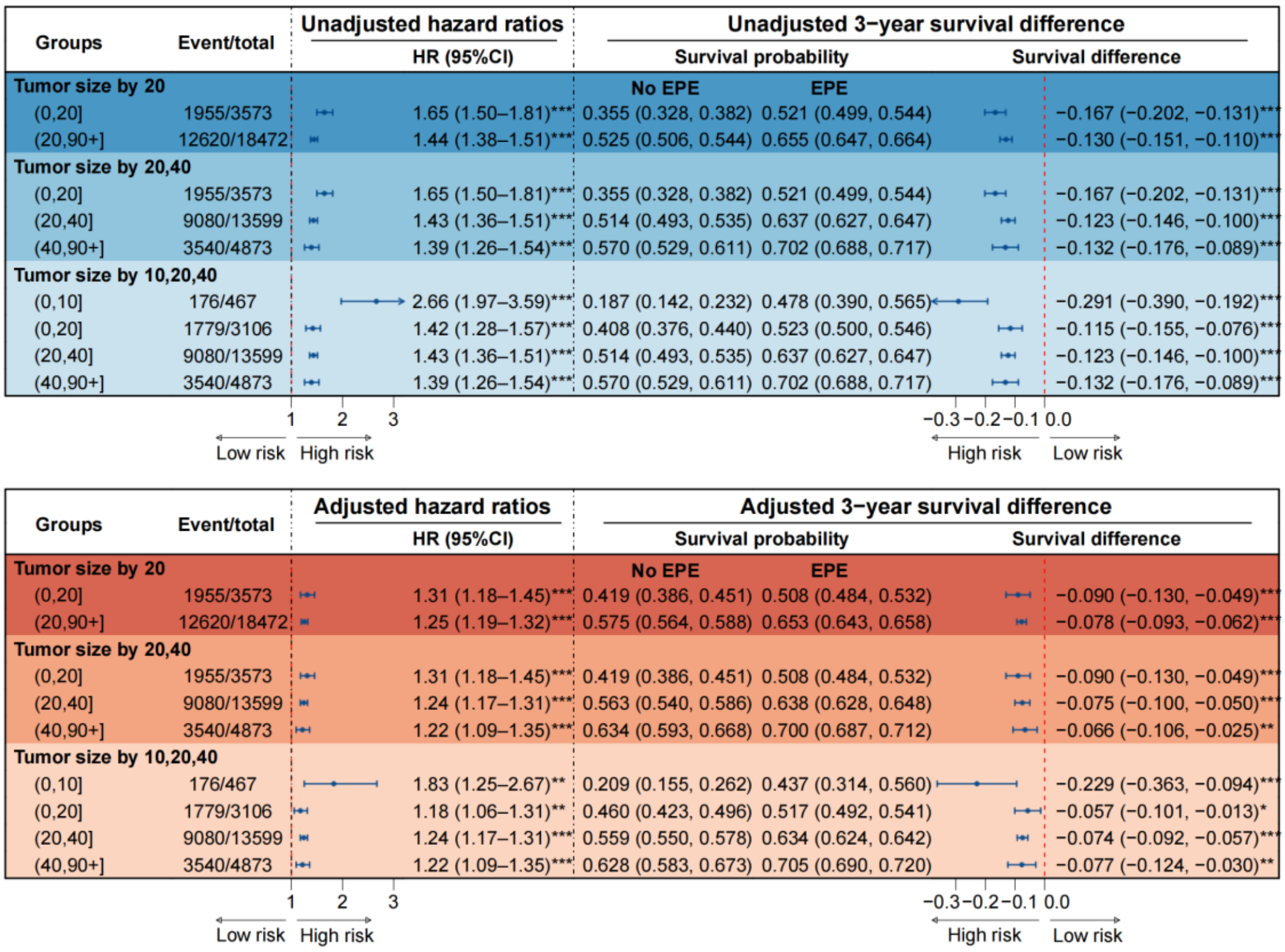

Supplementary Figure 4 Relative and absolute effects of EPE across different tumor size groups based on the Fine-Gray model.

Forest plots displaying unadjusted (upper panel) and adjusted (lower panel) subdistribution hazard ratios (left) and 3-year survival differences (right) for EPE versus no EPE within different tumor size strata. The adjusted analysis was derived from a multivariate Fine-Gray competing risk model adjusted for age, sex, race/ethnicity, marital status, household income, county type, year of diagnosis, grade, N stage, surgery, radiotherapy, and chemotherapy. ***p < 0.001, **p < 0.01, and *p < 0.05.

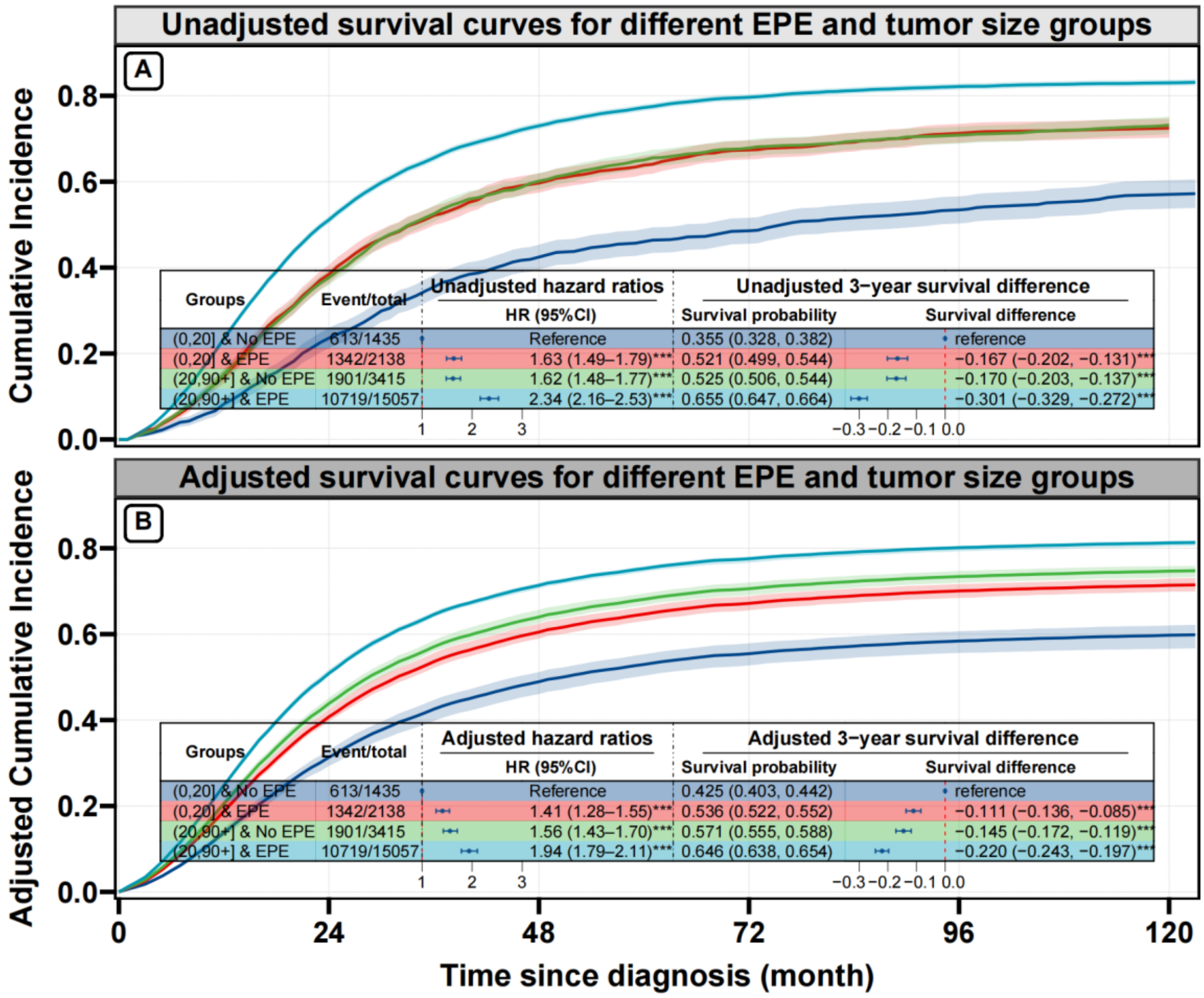

Supplementary Figure 5. Survival curves for different EPE and tumor size groups based on the Fine-Gray model.

(A) Unadjusted and (B) adjusted cumulative incidence curves for four patient groups stratified by EPE status and tumor size (≤20 mm vs. >20 mm). The adjusted curve was derived from a multivariate Fine-Gray competing risk model adjusted for age, sex, race/ethnicity, marital status, household income, county type, year of diagnosis, grade, N stage, surgery, radiotherapy, and chemotherapy. ***p < 0.001, **p < 0.01, and *p < 0.05.
